# Editorial: A year in review: discussions in obesity

**DOI:** 10.3389/fendo.2023.1215596

**Published:** 2023-05-31

**Authors:** Abbas Yadegar, Ali Nabavi-Rad, Dario Iafusco, Nahum Méndez-Sánchez

**Affiliations:** ^1^ Foodborne and Waterborne Diseases Research Center, Research Institute for Gastroenterology and Liver Diseases, Shahid Beheshti University of Medical Sciences, Tehran, Iran; ^2^ Department of Pediatric, University of Campania Luigi Vanvitelli, Naples, Italy; ^3^ Liver Research Unit, Medica Sur Clinic & Foundation, Mexico City, Mexico

**Keywords:** obesity, non-alcoholic fatty liver disease, gut microbiota, energy homeostasis, bariatric surgery

The prevalence of obesity has increased over the past decades and reached pandemic levels. Globally, obesity represents a serious threat to health due to the subsequent elevation in the risk of noncommunicable diseases (NCDs) such as type 2 diabetes, fatty liver disease, various cancers, hypertension, and dementia (1). Controlling the health and social burden of obesity and reducing its prevalence have become a priority for WHO. As the main cause of obesity is the long-term disparity between consumed and expended calories, WHO affirmed that a healthy diet and lifestyle with high physical activity should be cultivated for the entire population (2). In clinical practice for children and adolescents, body mass index (BMI) is commonly used for screening and diagnosing excess body fat and obesity (3). However, the critical involvement of biological factors in the pathogenesis of obesity such as genetic factors (4, 5), inheritance of an imbalance energy metabolism (6), and dysbiotic gut microbial compositions (7) is currently considered for the development of innovative therapeutic targets and diagnosis approaches. Current guidelines recommend a variety of interventions for different levels and grades of obesity including medical nutrition therapy, physical activity, psychological and behavioral interventions, pharmacotherapy, and bariatric surgery (8, 9). Therefore, researchers have expressed the intention of seeking novel obesity-associated risk factors, diagnostic methods, and therapeutic approaches.

In this case, the Research Topic of “*A year in review: Discussions in obesity*” appeared highly topical, presenting a discussion around popular spontaneous articles and articles in Research Topics from 2021 in obesity. In this collection, the aim was to highlight recent developments in the pathophysiology, diagnosis, and treatment of obesity and obesity-related diseases.

Mounting evidence supports the contribution of the gut microbiota in the etiology of obesity and metabolic disorders (10). In this regard, Cheng et al. reviewed the influence of alteration in the indigenous profile of the gut microbiota and metabolome on the disruption of energy homeostasis and the progression of obesity. This review article further emphasized the significant importance of microbiome-based therapeutics for reversing the increasing rate of obesity. Moreover, Juárez-Hernández et al. discussed current treatment approaches for non-alcoholic fatty liver disease (NAFLD) as an obesity-related complication. This study particularly focused on bariatric endoscopic and surgical therapies that could highly improve the metabolic profile and the quality of life, and also reduce the risk of cardiovascular diseases in NAFLD patients. Early together with liver fibrosis evaluation, these therapies could benefit non-responder patients to lifestyle modifications and pharmacological therapeutics.

NAFLD has become the most prevalent chronic liver disease with wide geographic variation across the world (11). There are currently several challenges in NAFLD diagnosis and risk stratification, particularly in the absence of histology. The liver biopsy as an invasive approach is sporadically applied and may encounter sampling error and lack inter-rater reliability. Therefore, non-invasive methods are crucial strategies for identifying potential candidates and tracking patient response to pharmacologic treatments (12). To this end, Hao et al. examined serum homocysteine level and body composition as potential markers for NAFLD diagnosis in Chinese healthcare workers. Evaluating a total of 4028 subjects, this study reported that BMI, waistline, neck-circumference, abdominal visceral fat area (VFA), total cholesterol, triglycerides, high-density lipoprotein cholesterol (HDL-C), glucose, and homocysteinemia were elevated in NAFLD patients, compared to healthy controls. Finally, Hao et al. concluded that skeletal muscle content, neck circumference, and hyperhomocysteinemia could be associated with the incidence of NAFLD and considered non-invasive diagnostic markers.

Bariatric surgery is proposed as the most effective treatment for severe obesity with longer life expectancy than conventional bariatric care (13). Han et al. investigated whether VFA might be a risk factor for early postoperative complications in obese patients undergoing bariatric surgery. Examination of a total of 152 patients at a tertiary university hospital presented a higher incidence of early postoperative complication for high-VFA patients, compared to low-VFA patients. This study, therefore, concluded that a high VFA level independently increases the risk of early postoperative complications after bariatric surgery and VFA concentration should be considered in preoperative assessments.

In conclusion, contributions to this Research Topic presented key factors contributing to the incidence, progression, and treatment of obesity and obesity-related diseases. Nevertheless, obesity is still a challenging field of research, which includes many unknown mechanisms in the pathophysiology of obesity and obesity-related disorders as well as their interactions with the gut microbiota. Resolving these challenges could pave the way for the development of novel diagnosis and treatment strategies.

## Author contributions

AN-R wrote the first draft of the manuscript. AY, DI, and NM-S critically revised the manuscript. All authors read and approved the submitted version.
